# Continuous versus discrete data analysis for gait evaluation of horses with induced bilateral hindlimb lameness

**DOI:** 10.1111/evj.13451

**Published:** 2021-06-23

**Authors:** Ineke H. Smit, Elin Hernlund, Harold Brommer, P. René van Weeren, Marie Rhodin, Filipe M. Serra Bragança

**Affiliations:** ^1^ Department of Clinical Sciences Faculty of Veterinary Medicine Utrecht University Utrecht The Netherlands; ^2^ Department of Anatomy, Physiology and Biochemistry Swedish University of Agricultural Sciences Uppsala Sweden

**Keywords:** clinical, data analysis, gait analysis, horse, kinematics

## Abstract

**Background:**

Gait kinematics measured during equine gait analysis are typically evaluated by analysing (asymmetry‐based) discrete variables (eg, peak values) obtained from continuous kinematic signals (eg, timeseries of datapoints). However, when used for the assessment of complex cases of lameness, such as bilateral lameness, discrete variable analysis might overlook relevant functional adaptations.

**Objectives:**

The overall aim of this paper is to compare continuous and discrete data analysis techniques to evaluate kinematic gait adaptations to lameness.

**Study design:**

Method comparison.

**Methods:**

Sixteen healthy Shetland ponies, enrolled in a research programme in which osteochondral defects were created on the medial trochlear ridges of both femurs, were used in this study. Kinematic data were collected at trot on a treadmill before and at 3 and 6 months after surgical intervention. Statistical parametric mapping and linear mixed models were used to compare kinematic variables between and within timepoints.

**Results:**

Both continuous and discrete data analyses identified changes in pelvis and forelimb kinematics. Discrete data analyses showed significant changes in hindlimb and back kinematics, where such differences were not found to be significant by continuous data analysis. In contrast, continuous data analysis provided additional information on the timing and duration of the differences found.

**Main limitations:**

A limited number of ponies were included.

**Conclusions:**

The use of continuous data provides additional information regarding gait adaptations to bilateral lameness that is complementary to the analysis of discrete variables. The main advantage lies in the additional information regarding time dependence and duration of adaptations, which offers the opportunity to identify functional adaptations during all phases of the stride cycle, not just the events related to peak values.

## 1 INTRODUCTION

Currently, quantitative gait analysis systems for clinical lameness evaluations in horses rely on the detection of movement asymmetries between left and right.^1^ Typically, 3‐dimensional (3D) kinematic signals are recorded, separated into multiple continuous 2D angle‐time or displacement‐time signals and then further analysed by extracting single (peak) values. Using this approach, the horses’ complex motion pattern is reduced to a manageable amount of scalar, time discrete variables.

Several kinematic and kinetic differences between the locomotion of healthy and unilaterally lame horses have already been identified.^1^ These include decreased vertical displacement of the head, withers and/or pelvis during the stance phase of the lame limb,^2^, ^3^, ^4^ increased upward movement of the tuber coxae before touchdown of the affected limb (hip hike)^5^ and reduced peak vertical force (PVF) of the affected limb,^6^, ^7^ all resulting in movement asymmetry. However, these discrete variables represent only a small part of the horse's movement and when an asymmetric pattern is absent, such as in cases of bilateral lameness,^8^ analyses based solely on such discrete variables may be insufficient to discriminate between healthy and lame horses.

The reliance on discrete variables to identify gait adaptations to lameness has three limitations. Firstly, adaptations may occur over phases of the stride that cannot be described by single discrete variables. Secondly, the timing of single values can differ between sides without changing in amplitude. And thirdly, discrete variables are not necessarily independent and analysing them as such may result in bias.^9^ To overcome these limitations, continuous data analysis techniques,^10^ such as statistical parametric mapping (SPM),^9^ have been developed. To assess the value of continuous data analysis for identifying functional adaptations to lameness in general and more specifically to bilateral hindlimb lameness in equine locomotion analysis, a comparison of kinematic findings retrieved from continuous versus discrete analyses is warranted.

The purpose of the current study was to compare results from continuous and discrete data analysis techniques to evaluate kinematic adaptations to induced bilateral hindlimb lameness. We hypothesised that continuous data analysis techniques would provide more detailed information about functional kinematic adaptations compared to the analysis of discrete values.

## 2 MATERIALS AND METHODS

### 2.1 Animals

Sixteen sound Shetland ponies were used in this study. All ponies were enrolled in an articular cartilage repair study in which they underwent a surgical intervention to create osteochondral defect bilaterally on the medial trochlear ridges of both femurs and treated by the implantation of a bio‐engineered scaffold.^11^ All were mares, with an age distribution of 4‐12 years and a mean ± SD body mass of 169 ± 29 kg.

### 2.2 Data collection

Prior to the experiment, the ponies were accustomed to treadmill exercise.^12^ Kinematic data were recorded using six infra‐red three‐dimensional (3D) optical motion capture (OMC) cameras (Qualisys AB, Motion Capture Systems) that registered the positions of 28 skin mounted spherical reflective markers (19‐24 mm) at 200 Hz. For detailed marker placement, see Figure S1. Data collection lasted 30 s for each trial at trot on a treadmill after a warm‐up period at walk and trot. Measurements were performed at the individually preferred trotting speed for each pony, based on visual assessment of locomotion regularity.^12^ Subsequent measurements were speed matched, ensuring control over speed along all timepoints. The ponies were measured at three timepoints: prior to the surgical intervention at baseline (T0), and at 3 months (T1) and 6 months (T2) after surgical intervention.

### 2.3 Data processing

The reconstruction of the 3D coordinates of each marker was automatically calculated by using motion capture software (QTM^a^, version 2.9). Each marker was identified and labelled using an automated model and manually checked. Raw data consisting of the 3D data of the designated markers were exported to Matlab (version 2019b) (The MathWorks Inc, Natick, Massachusetts, USA) for further analysis using custom written scripts. Stride segmentation was performed based on the maximal vertical position of the tuber sacrale before maximal protraction of the left hindlimb. All signals were high‐pass filtered using a fourth‐order Butterworth filter with the cut‐off frequency adjusted based on the stride frequency of the individual ponies.^13^ Strides with excessive head movement (two SD’s from the trial mean) were automatically removed. For further analysis, the first 20 strides of each trial were selected for each pony. Bone segments were formed based on marker locations and angles between these segments were calculated for each stride. See Table 1 for variable definitions. The data were exported as discrete variables (ie, minima, maxima, and range of motion (ROM)) for discrete data analysis and exported as a timeseries of 101 datapoints per stride for continuous data analysis.

**TABLE 1:** Overview of the kinematic variables used. Units are given in millimetres (mm) for displacement variables and degrees (deg) for variables expressed in angles

Name	Units	Description	Anatomical landmarks
*Body*			
MinDiff	mm	Absolute difference between the left and right stride half‐cycle in minimum vertical position	Head: poll Withers: T8 Sacrum: Tuber sacrale
MaxDiff	mm	Absolute difference between the left and right stride half‐cycle in maximum vertical position	Head: poll Withers: T8 Sacrum: Tuber sacrale
Pelvis roll	deg	Rotation of the pelvis around the longitudinal axis of the horse	Tuber sacrale, left/right tuber coxae
Pelvis pitch	deg	Rotation of the pelvis around the transversal axis	Tuber sacrale, left/right tuber coxae
Pelvis yaw	deg	Rotation of the pelvis around the vertical axis	Tuber sacrale, left/right tuber coxae
Back flexion‐extension	deg	Rotation of the back around the transverse axis, with T15 as the point of rotation.	T8, T15, tuber sacrale
Back lateral bending	deg	Rotation of the back around the vertical axis, with T15 as the point of rotation.	T8, T15, tuber sacrale
*Limbs*			
Protraction	deg	Sagittal movement of the whole limb	Elbow, hoof
Fetlock extension	deg	Sagittal rotation around the fetlock joint	Proximal and distal end of metacarpal/metatarsal bone, hoof

### 2.4 Data analysis

For the analysis of discrete variables, stride‐level data were analysed in Open software R (version 3.3.1) (R‐studio, Boston, Massachusetts, USA), using package lme4 (version 1.1‐15) for mixed modelling. In each linear mixed model (LMM), random effect was “pony” and “timepoint” was used as the fixed effect. The dependent variables were investigated for a transformation close to normality using probability plotting and examining for skewness and kurtosis. When nonnormally distributed variables were found, these variables were transformed using the Box‐Cox method. The model estimates were represented as least squares means and confidence intervals.

For SPM analysis of the kinematic data, the mean value of the curves was subtracted for each timepoint to compensate for possible marker placement errors between trials. The normalised stride values were assembled into 20*101*1 vector fields (20 strides, 101 data points, 1 dimension per data point) for each joint, timepoint, and pony. The open source spm1d package (version M.0.4.1, Pataky, 2012) was used to conduct the SPM analysis in Matlab. Repeated measures ANOVAs were performed to compare kinematics between the three timepoints. If there were significant results, post hoc paired *t* tests were done to determine which timepoints were different. For both the SPM and discrete value analyses, significance was set at *P* value < .05, and *P* values were adjusted for multiple comparisons using the Benjamin‐Hochberg procedure.^14^

## 3 RESULTS

Two ponies were lost from the study: one due to severe lameness, another because no baseline measurement was recorded. Also, due to a misplaced marker, trials for forelimb kinematics were removed for one pony. The mean ± SD trotting speed was 2.18 ± 0.16, 2.21 ± 0.15 and 2.21 ± 0.16 m/s for T0, T1 and T2, respectively. At all timepoints the mean stride duration was 0.54 ± 0.01 s.

### 3.1 Discrete variable analysis

Discrete variable findings for the differences between the three timepoints can be found in Table 2. In terms of symmetry parameters, no significant differences (*P* > .05) were found between timepoints, except for the maxDiff of the withers at T1 (*P* < .001), which increased by 0.7 mm. For other kinematic variables, represented as the percentage of change in ROM at T1 and T2 compared to T0, significant differences between timepoints included a decrease in fetlock extension (−4.5% to −8.1%, *P* < .001) of the forelimbs, back flexion‐extension (−9.5% to −10.5%, *P* < .001) and pelvis pitch (−12.2% to −18.6%, *P* < .001). Significant increases were found in lateral bending of the back (6.3% to 8.8%, *P* < .001), pelvis yaw (7.4% to 11.6%, *P* < .001), pelvis roll (8.2% to 12.6%, *P* < .001) and protraction of the forelimbs (3.0% to 5.2%, *P* < .001). The differences in pelvis pitch and forelimb fetlock extension significantly increased over time. In contrast, the differences in pelvis yaw and forelimb protraction peaked at T1 and decreased again at T2 in relation to T0.

**TABLE 2:** Linear mixed model (discrete variable analysis) results

			T0		T1		T1‐T0			T2		T2‐T0		
Variable	Units	Location	Estimate	SE	Estimate	SE	Difference	% Difference	*P* value	Estimate	SE	Difference	% Difference	*P* value
MinDiff	mm	Head	6.7	0.7	6.8	0.7	0.1		>.9	6.8	0.7	0.1		>.9
		Withers	3.9	0.5	4.0	0.5	0.1		>.9	3.6	0.5	−0.3		.6
		Pelvis	4.2	0.4	4.7	0.4	0.5		.2	4.5	0.4	0.4		.5
MaxDiff	mm	Head	7.9	1.1	7.5	1.1	−0.3		>.9	7.9	1.1	0.0		>.9
		Withers	2.7	0.4	3.4	0.4	0.7		<.001	2.8	0.4	0.1		>.9
		Pelvis	5.4	1.1	5.4	1.1	0.0		>.9	5.0	1.1	−0.4		.5
Vertical ROM	mm	Head	39.3	1.5	38.8	1.5	0.4	−1.1	>.9	39.8	1.5	−0.5	1.3	.8
		Withers	34.4	1.4	35.2	1.4	0.9	2.5	.07	36.7	1.4	2.3	6.8	<.001
		Pelvis	39.5	1.7	39.8	1.7	0.3	0.8	.8	39.4	1.7	−0.1	−0.2	>.9
Flexion—extension	deg	Back	4.6	0.2	4.1	0.2	−0.5	−10.5	<.001	4.1	0.2	−0.4	−9.5	<.001
Lateral bending	Back	6.5	0.4	7.1	0.4	0.6	8.8	<.001	6.9	0.4	0.4	6.3	<.001
Roll	deg	Pelvis	5.0	0.3	5.4	0.3	0.4	8.2	<.001	5.6	0.3	0.6	12.6	<.001
Pitch	Pelvis	7.3	0.4	6.4	0.4	−0.9	−12.2	<.001	6.0	0.4	−1.4	−18.6	<.001
Yaw	Pelvis	3.7	0.3	4.1	0.3	0.4	11.6	<.001	3.9	0.3	0.3	7.4	<.001
Fetlock extension ROM	deg	LF	−35.0	0.6	−33.4	0.6	1.6	−4.5	<.001	−32.7	0.6	2.3	−6.6	<.001
		RF	−35.2	0.6	−33.0	0.6	2.2	−6.3	<.001	−32.3	0.6	2.9	−8.1	<.001
		LH	−39.0	1.0	−39.7	1.0	−0.7	1.9	<.001	−38.7	1.0	0.3	−0.7	.5
		RH	−38.5	0.9	−37.3	0.9	1.2	−3.0	<.001	−37.8	0.9	0.7	−1.9	<.001
Protraction ROM	deg	LF	38.1	0.7	40.0	0.7	2.0	5.2	<.001	39.4	0.7	1.4	3.6	<.001
		RF	38.1	0.7	40.0	0.7	1.9	4.9	<.001	39.3	0.7	1.2	3.0	<.001
		LH	37.8	0.8	39.5	0.8	−0.2	−0.6	.4	37.5	0.8	−0.2	−0.7	.3
		RH	37.3	0.7	28.8	0.7	0.0	−0.1	>.9	37.4	0.7	0.1	0.3	.9

LF = left forelimb; RF = right forelimb; LH = left hindlimb; RH = Right hindlimb; ROM = range of motion. All angle values describe the range of motion. For trunk and hindlimb kinematics: N = 14, for forelimb kinematics: N = 13. Units are given in millimetres (mm) for displacement variables and degrees (deg) for variables expressed in angles.

### 3.2 Continuous data analysis

**FIGURE 1:**
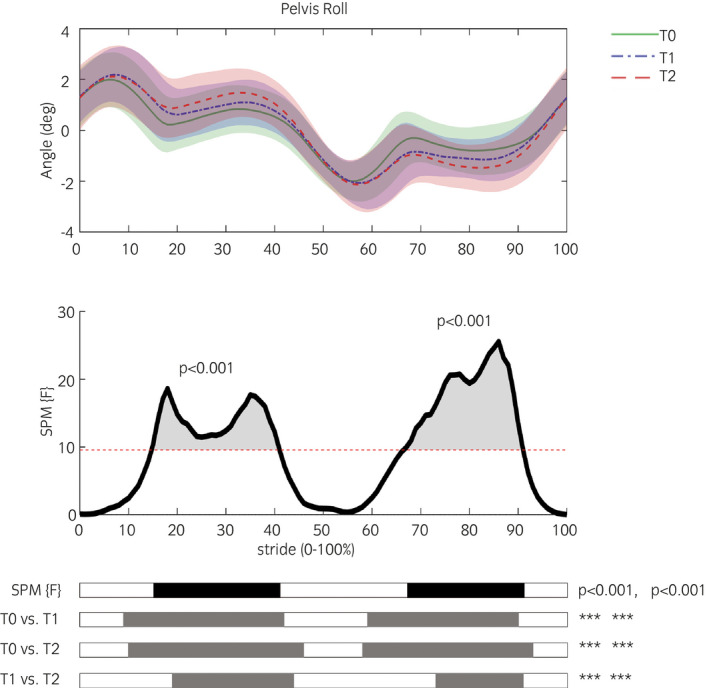
Example of summarised presentation of statistical parametric mapping (SPM) results. The upper graph shows the mean kinematic pelvis roll angle at the three timepoints. The middle graph shows the SPM {F} statistic as a function of the gait cycle. The critical threshold (red dashed line) was exceeded between 15%‐42% and 67%‐91% of the gait cycle. Lower black bars represent a simplified visualisation of the significant areas indicated by the SPM {F} statistic. Grey bars represent a simplified visualisation of the post hoc paired analysis of the differences between all timepoints (ie, post hoc paired t test (SPM {t}), *a* = 0.0036). ^*^ *P* < .05, ^**^ *P* < .01, ^***^ *P* < .001

**FIGURE 2:**
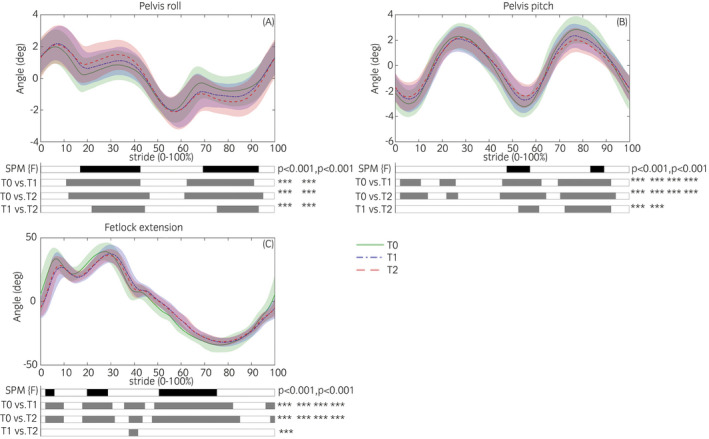
Each graph shows the mean kinematic signal of T0 (green‐solid), T1 (blue‐dashed) and T2 (red‐dashed) at the level of each joint. A, Pelvis roll angle, demonstrating a change outside of peak values. B, Pelvis pitch angle, demonstrating changes on peak values. C, Fetlock extension angles for the right forelimb, demonstrating a time shift of the complete curve. Black bars indicate gait phases during which the SPM {F} statistic exceeded the critical threshold. Grey bars indicate gait phases during which the SPM {t} statistic exceeds the critical threshold in the post hoc analysis (ie, post hoc paired *t* test, *a* = 0.0036). ^*^ *P* < .05, ^**^ *P* < .01, ^***^ *P* < .001

Figure 1 illustrates SPM findings. Representative SPM results are shown in Figure 2, further SPM figures can be found in Figures [Link], [Link], [Link], [Link]. There were no significant differences between the left and right step (Figures S4 and S5). Between timepoints, significant differences were found in pelvis kinematics (Figure 2A,B), where both at T1 and T2 in relation to T0, pelvis roll (Figure 2A) increased during the stance phase (*P* < .001) and pelvis pitch (Figure 2B) decreased at its extremes during both the stance and swing phase of the right step (*P* < .001). Changes in limb kinematics appeared in the forelimbs (Figure 2C), where the curve was significantly delayed with regard to the stride split at T1 and T2 in relation to T0 (*P* < .001).

## 4 DISCUSSION

The purpose of the current study was to compare continuous and discrete data analysis techniques to evaluate gait adaptations in cases of induced bilateral hindlimb lameness. With the use of the discrete variable analysis, more variables were found to change significantly compared to continuous data analysis. However, changes outside the peak value areas and the duration of the changes are not taken into account using discrete variables alone, whereas the continuous data analysis considered both.

Analysis of trunk kinematics indicated there was no change in upper body vertical movement, except for the maxDiff of the withers, which was, however, of minor magnitude that is deemed not clinically relevant.^15^ Both types of analysis identified comparable adaptations in pelvis pitch and back flexion‐extension kinematics. The change in back flexion‐extension was only significant for the discrete variable analysis but a trend was present in the continuous data. The curves for these movements are all sinusoidal and the adaptations are largest at the peaks (Figure 2B). Discrete variable analysis indicated considerable increases in pelvis yaw and back lateral bending, which were not identified using continuous data analysis. For pelvis roll kinematics, both types of analyses indicated an increase in pelvis roll angles. The continuous data analysis showed that time wise the difference was largest from mid‐ to late stance of each hindlimb (Figure 2A), the discrete analysis only identified the significant difference in ROM. The analysis of continuous data hence provides additional information about the moment and duration of the differences during the stride, which can help us better understand and explain the dynamics of gait adaptations to lameness.

In hindlimb fetlock extension angles, small but significant differences were identified using discrete variable analysis, which were not detected by continuous data analysis. Both methods suggested a decrease in protraction ROM and an increase in fetlock extension ROM for both forelimbs. The SPM results additionally showed the presence of significant delays in the timing of the sagittal plane movement of the forelimbs with regard to stride segmentation (Figure 2C). In this study the stride segmentation is based on maximal vertical displacement of the sacrum, which is tightly related to the timing of hindlimb kinematics.^16^ Therefore, it is possible that forelimb kinematics are not delayed, but that the hindlimb placement is advanced, resulting in earlier support of the trunk during the stride cycle, which is consistent with findings of studies on unilateral lameness.^2^

There are several explanations for the differences in findings between continuous and discrete data analysis. Firstly, fewer parameters were found to be significant using SPM compared to LMM. This is concordance with earlier studies comparing discrete to continuous data analysis of under hoof ground reaction forces.^17^, ^18^ SPM uses random field theory^19^ to ensure a tight control of alpha. This may have resulted in fewer type I errors made compared to LMM,^9^ which assumes a point‐process Gaussian variance model.^20^ Hobbs et al^17^ pointed out that, due to this tight control of alpha in SPM analysis, reaching significance may not be as important as understanding the clinical implications of functional adaptations. Secondly, with discrete data analysis, only single points on the extremes of the signal were selected for further analysis. Failure to test differences throughout the complete signal means that relevant adaptations that occurred outside of these extremes might be missed. And lastly, both amplitude and timing of events may have varied between timepoints and individuals. These amplitude and timing variations can be influenced by factors such as skin displacement artefacts,^21^ placement of markers^22^ and further habituation to the measurement situation.^12^ With shifts in timing of events between trials and individuals, the peak angles may be cancelled out when summarising continuous signals. Thus, with variability of movement and fluctuations in timing and amplitude, peak angles become less evident when using continuous data, but not with discrete variables.

The analysis of continuous data may hold additional advantages regarding the evaluation of equine lameness. Firstly, the analysis of continuous signals may help to further differentiate during the lameness exam. This has been shown for equine gait kinetics, where for instance palmar foot syndrome and mild tendinopathy were associated with changes in the shape of the vertical ground reaction force curve, rather than simply a reduction of PVF in the lame limb(s).^23^ Hobbs et al^17^ also used SPM on the individual kinetic pattern and found subtle asymmetries in the sound horse. They suggest that, if the periods of asymmetry can be related to specific events in the stride cycle, it may help the interpretation of their functional significance. However, subtle asymmetries might be lost when data are averaged over a group of horses, which indicates the importance of evaluating horses on an individual basis.^17^ In case of knee osteoarthritis in humans, it has been shown that hip and knee rotation patterns differ between medial and lateral knee osteoarthritis^24^ and both joint moment and joint angle patterns change with the severity of the disease.^25^ Thus, the timing of the change can be related to the function of the affected structure and may therefore help to localise the pain. Changed joint angle patterns related to decreased joint loading have already been used to alter walking patterns,^26^ showing potential for both diagnosis and rehabilitation purposes. Changes of this nature should be detectable using continuous data analysis techniques but are overlooked when only using discrete variables. Secondly, results of kinematic analyses using continuous data may better resemble the daily practice of evaluation of locomotion during lameness exams compared to considering only discrete variables. When assessing individual horses, veterinarians usually follow a structured protocol of observation: systematically assessing the overall movement pattern, then focus on the region of interest (such as the head or pelvis) and afterwards determine at which point in the gait cycle the most obvious abnormality is visible. Further investigations are needed to determine to which extent continuous data analysis techniques agree with clinical assessments of movement patterns during lameness evaluations.

In conclusion, this study has shown that the use of continuous data provides additional information regarding gait adaptations to bilateral lameness that is complementary to the analysis of discrete variables. The main asset lies in the additional information regarding time dependence and duration of adaptations. This offers opportunities to identify functional adaptations during all phases of the stride cycle, instead of only during events related to peak values.

## CONFLICT OF INTERESTS

No competing interests have been declared.

## AUTHOR CONTRIBUTIONS

I. Smit and F. Serra Bragança contributed to data collection, data processing and statistics. E. Hernlund contributed to the statistics. F. Serra Bragança, H. Brommer and R. van Weeren contributed to planning the experiment. All authors contributed to the preparation of the manuscript and approved the final version.

## ETHICAL ANIMAL RESEARCH

The study was approved by the Ethical Committee of Utrecht University (Approval number AVD108002015307 WP23).

### PEER REVIEW

The peer review history for this article is available at https://publons.com/publon/10.1111/evj.13451.

## Supporting information

Fig S1

Fig S2

Fig S3

Fig S4

Fig S5

Chinese Summary

## Data Availability

The complete data set used in this study including raw optical motion capture (OMC) data and processed OMC data can be accessed using the following doi https://doi.org/10.24416/UU01‐IYO9I8.
