# Supplementary material for: Continuous versus discrete data analysis for gait evaluation of horses with induced bilateral hindlimb lameness
Source: Equine Vet J. 2021 Jun 23;54(3):626–33. doi: 10.1111/evj.13451 (PMC9290451; doi:10.1111/evj.13451)

Equine Veterinary Journal BEVA

**Figure S1:** One of the study subjects, equipped with 28 reflective markers, at trot. The markers used for the analysis were: poll, withers, dorsal spinal process of T15, tuber sacrale, left/right tuber coxae, left/right spina scapulae, left/right greater tubercle of the humerus, left/right lateral aspect of the carpus, left/right- front/hind lateral aspect of the fetlock, left/right- front/hind lateral aspect of the proximal dorsal hoof wall, left/right trochanter major of the femur and left/right lateral aspect of the tarsus.

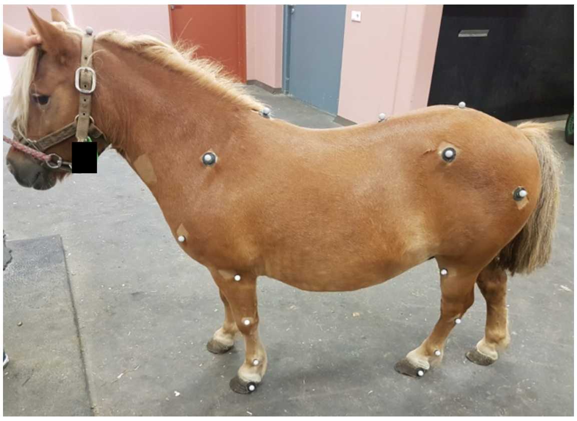

Supplement: Supplementary file 1 — Fig S1 [file EVJ-54-626-s002.pdf]
