# Supplementary material for: Continuous versus discrete data analysis for gait evaluation of horses with induced bilateral hindlimb lameness
Source: Equine Vet J. 2021 Jun 23;54(3):626–33. doi: 10.1111/evj.13451 (PMC9290451; doi:10.1111/evj.13451)

Equine Veterinary Journal

Figure S2: Mean angle-time trajectories and standard deviation clouds for body kinematic variables between timepoints. Each graph shows the mean kinematic signal of T0 (green-solid), T1 (blue-dashed) and T2 (red-dashed) at the level of each joint. Black bars indicate gait phases during which the SPM {F} statistic exceeded the critical threshold. Grey bars indicate gait phases during which the SPM {t} statistic exceeds the critical threshold in the post-hoc analysis (i.e. post-hoc paired t-test, a = 0.0036). * p<0.05, ** p<0.01, *** p<0.001

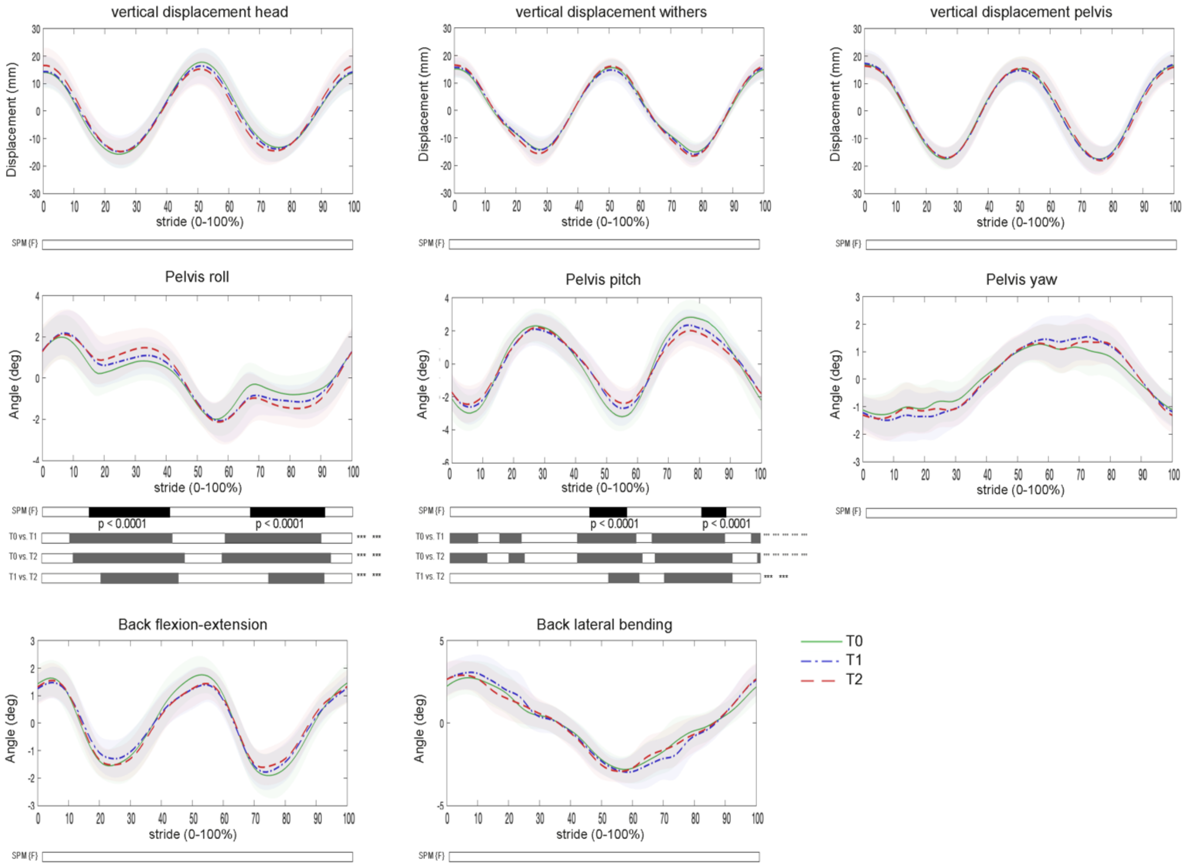

Supplement: Supplementary file 2 — Fig S2 [file EVJ-54-626-s005.pdf]
