# Supplementary material for: Continuous versus discrete data analysis for gait evaluation of horses with induced bilateral hindlimb lameness
Source: Equine Vet J. 2021 Jun 23;54(3):626–33. doi: 10.1111/evj.13451 (PMC9290451; doi:10.1111/evj.13451)

Equine Veterinary Journal BEVA

Figure S4: Mean angle-time trajectories and standard deviation clouds for body kinematic variables for the left (green solid) and right step (red dashed) for each timepoint separately (top: T0, middle: T1 and bottom: T2). The stride is defined as maximal vertical position of the tuber sacrale before left hind (LH) impact to the next maximal position before LH impact. The left step is defined as 0-50% of the stride, the right step as 50-100% of the stride. For lateral bending of the back, pelvis roll and pelvis yaw, right step values are multiplied by -1 to be able to make a direct comparison. For the left and right limb pairs, the whole stride is compared from LH impact to LH impact for left sided limbs and right hind (RH) impact to RH impact for right sided limbs.

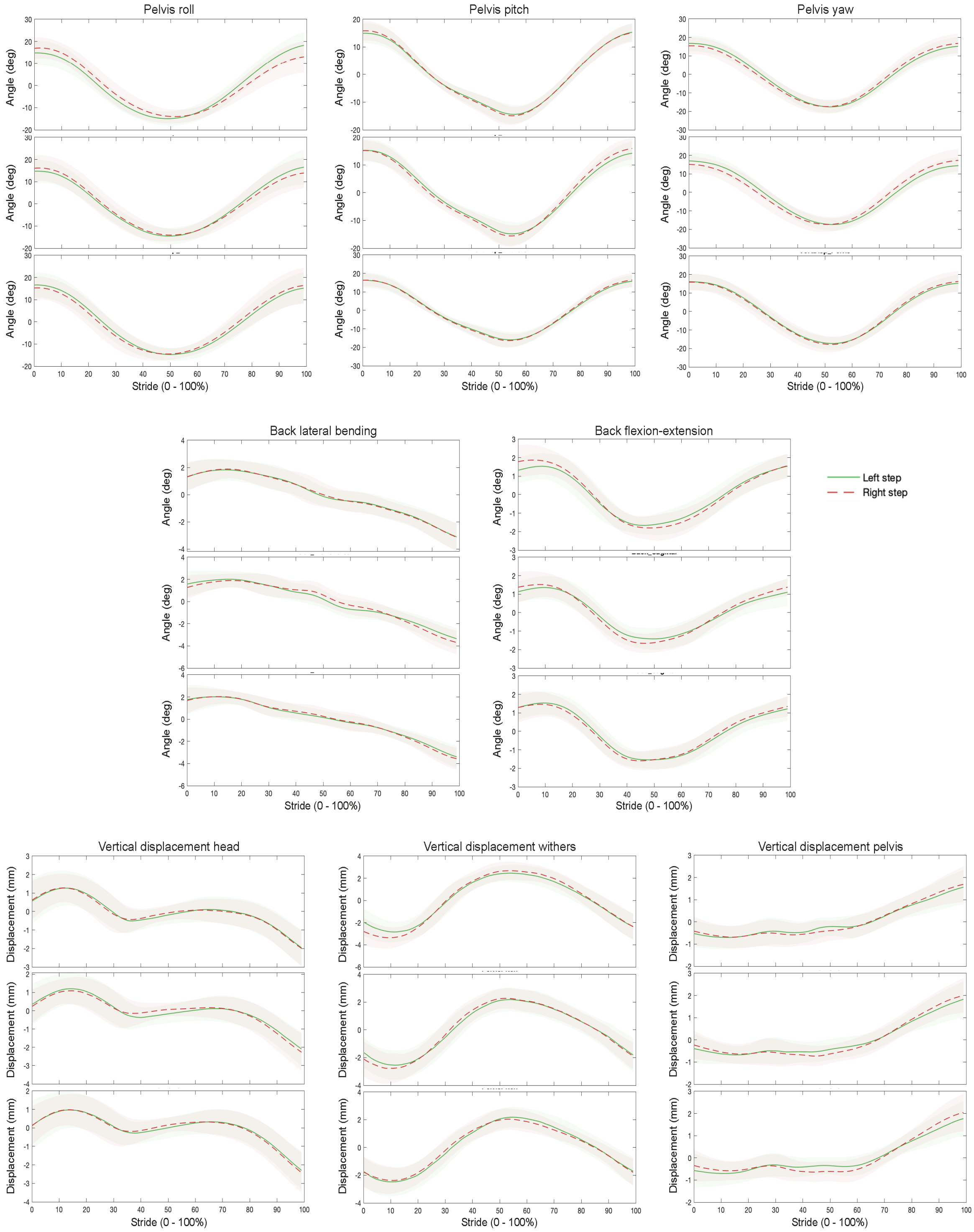

Supplement: Supplementary file 4 — Fig S4 [file EVJ-54-626-s006.pdf]
