# Supplementary material for: Continuous versus discrete data analysis for gait evaluation of horses with induced bilateral hindlimb lameness
Source: Equine Vet J. 2021 Jun 23;54(3):626–33. doi: 10.1111/evj.13451 (PMC9290451; doi:10.1111/evj.13451)

Equine Veterinary Journal BEVA

Figure S5: Mean angle-time trajectories and standard deviation clouds for limb kinematic variables for the left (green solid) and right stride (red dashed) for each timepoint separately (top: T0, middle: T1 and bottom: T2). The left stride is defined as maximal vertical position of the tuber sacrale before left hind (LH) impact to the next maximal position before LH impact for the left limbs. The right stride is defined as maximal vertical position of the tuber sacrale before right hind (RH) impact to the next maximal position before RH impact for the right limbs.

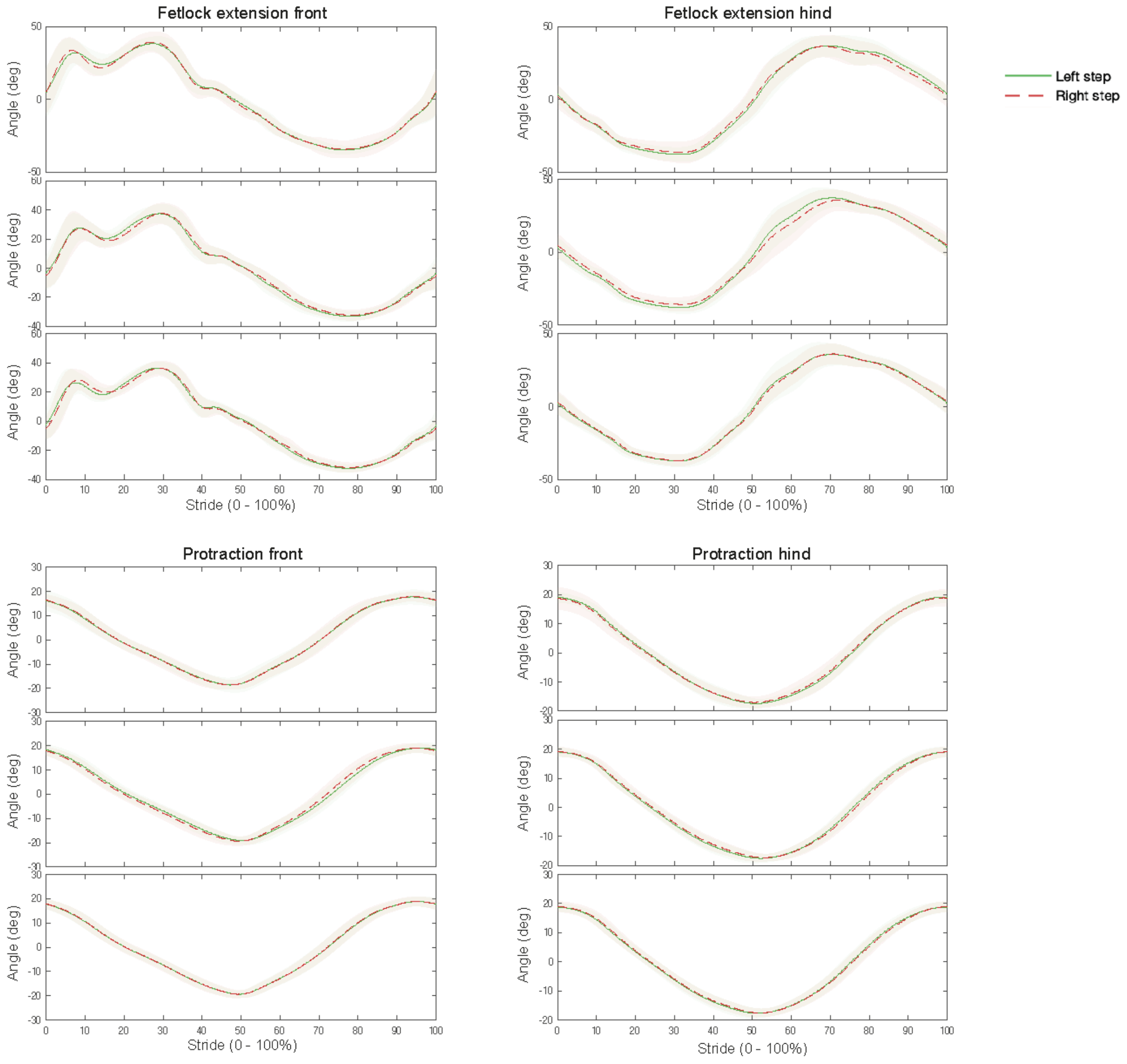

Supplement: Supplementary file 5 — Fig S5 [file EVJ-54-626-s001.pdf]
