# Supplementary material for: Continuous versus discrete data analysis for gait evaluation of horses with induced bilateral hindlimb lameness
Source: Equine Vet J. 2021 Jun 23;54(3):626–33. doi: 10.1111/evj.13451 (PMC9290451; doi:10.1111/evj.13451)
Supplement: Supplementary file 6 — Chinese Summary [file EVJ-54-626-s004.docx]

诱导马匹双侧后肢跛行后，使用连续与离散数据分析进行步态评价

Ineke H. Smit*^1^, Elin Hernlund^2^, Harold Brommer^1^, P. René van Weeren^1^, Marie Rhodin^2^ and Filipe M. Serra Bragança^1^

^1^乌得勒支大学，兽医学院，临床科学系; 乌得勒支，荷兰

^2^瑞典农业科学大学，解剖、生理和生物化学系；乌普萨拉，瑞典

*通讯作者：I.h.smit@outlook.com

**总结**

**背景：**马的步态分析中，使用步态运动学通常通过分析（基于不对称性）离散变量（如峰值）来评估的，这些离散变量来自于连续的运动信号（如根据时间序列的数据点）。然而，当用于评估复杂的跛行情况，如双侧跛行时，离散变量分析可能会忽略相关的功能适应性。

**目的：**本文的总体目标是比较连续和离散的数据分析技术，以适当评估跛行的运动步态。

**研究设计：**方法比较。

**方法:** 本研究募集了16匹健康的舍特兰矮种马，人工制造了双侧股骨滑车内侧脊处骨软骨缺损。在术前、术后3个月和6个月分别在跑步机上收集运动学数据。使用统计参数图和线性混合模型，比较不同时间点之间的运动学变量。

**结果:** 连续和离散的数据分析都确定了骨盆位置和前肢位置运动学的变化。离散数据分析显示，后肢和背部运动学有显著变化，而连续数据分析发现这些差异并不显著。相反，连续数据分析提供了关于发现差异的时间和持续时间的额外信息。

**主要限制:** 使用矮马，数量有限。

**结论:** 连续数据的使用提供了双侧跛行马匹步态适应性的额外信息，这是对离散变量分析的补充。主要的优点是关于适应性的时间依赖性和持续时间的额外信息，这为识别整个步幅周期的功能性适应提供了机会，而不仅仅识别与峰值相关的指标。

**关键词:** 马，步态分析，临床，运动学，数据分析
